# Virological and Serological Findings in *Rousettus aegyptiacus* Experimentally Inoculated with Vero Cells-Adapted Hogan Strain of Marburg Virus

**DOI:** 10.1371/journal.pone.0045479

**Published:** 2012-09-17

**Authors:** Janusz T. Paweska, Petrus Jansen van Vuren, Justin Masumu, Patricia A. Leman, Antoinette A. Grobbelaar, Monica Birkhead, Sarah Clift, Robert Swanepoel, Alan Kemp

**Affiliations:** 1 Center for Emerging and Zoonotic Diseases, National Institute for Communicable Diseases of the National Health Laboratory Service, Sandringham, South Africa; 2 Division Virology and Communicable Disease Surveillance, School of Pathology, University of the Witwatersrand, Johannesburg, South Africa; 3 Southern African Center for Infectious Disease Surveillance, Morogoro, Tanzania; 4 University of Pretoria, Pretoria, South Africa; University of Pretoria, South Africa

## Abstract

The Egyptian fruit bat, *Rousettus aegyptiacus*, is currently regarded as a potential reservoir host for Marburg virus (MARV). However, the modes of transmission, the level of viral replication, tissue tropism and viral shedding pattern remains to be described. Captive-bred *R. aegyptiacus*, including adult males, females and pups were exposed to MARV by different inoculation routes. Blood, tissues, feces and urine from 9 bats inoculated by combination of nasal and oral routes were all negative for the virus and ELISA IgG antibody could not be demonstrated for up to 21 days post inoculation (p.i.). In 21 bats inoculated by a combination of intraperitoneal/subcutaneous route, viremia and the presence of MARV in different tissues was detected on days 2–9 p.i., and IgG antibody on days 9–21 p.i. In 3 bats inoculated subcutaneously, viremia was detected on days 5 and 8 (termination of experiment), with virus isolation from different organs. MARV could not be detected in urine, feces or oral swabs in any of the 3 experimental groups. However, it was detected in tissues which might contribute to horizontal or vertical transmission, e.g. lung, intestines, kidney, bladder, salivary glands, and female reproductive tract. Viremia lasting at least 5 days could also facilitate MARV mechanical transmission by blood sucking arthropods and infections of susceptible vertebrate hosts by direct contact with infected blood. All bats were clinically normal and no gross pathology was identified on post mortem examination. This work confirms the susceptibility of *R. aegyptiacus* to infection with MARV irrespective of sex and age and contributes to establishing a bat-filovirus experimental model. Further studies are required to uncover the mode of MARV transmission, and to investigate the putative role of *R. aegyptiacus* as a reservoir host.

## Introduction

Marburg virus (MARV) and Ebola virus (EBOV) are non-segmented negative-strand RNA viruses of the family *Filoviridae*, causing a severe hemorrhagic fever (HF) syndrome in humans and non-human primates with high fatality [1]. The sporadic outbreaks of filovirus infections in humans are believed to result from contact with an infected animal and subsequent transmission between persons by direct contact with infected blood or body fluids [2]–[5]. Infected individuals succumbing to filovirus infection exhibit virus-mediated impairment of early innate immune responses allowing for rapid progression of filovirus infection [6]. The unavailability of antiviral therapy or approved vaccines, and the elusive nature of the spillover of filoviruses from a reservoir source to humans hamper countermeasures to effectively prevent the severe course of filovirus disease and transmission. The most pathogenic EBOV species in humans is *Zaire ebolavirus* with a case fatality rate (CFR) of up to 90%, followed by *Sudan ebolavirus* with a CFR of about 50% [1]. The first reported filovirus outbreak, involving MARV, occurred in Europe in 1967 with a CFR of 23% [7], [8]. All primary cases were laboratory workers who had close contact with blood and organs of African green monkeys (*Chlorocebus aethiops*) imported from Uganda. Experimental infection of *C. aethiops* and rhesus monkeys with MARV resulted in a fatal illness irrespective of route or dose of infection [9], indicating that these animals are not natural hosts of the virus. Between 1967 and 1998 Marburg HF was recognized only on three occasions, involving sporadic cases in South Africa in 1975 [10], and in Kenya in 1980 and 1987 [11], [12]. Large outbreaks of Marburg HF occurred in 1998–2000 in the Democratic Republic of the Congo [13], and in 2005 in Angola [14], [15]. Case fatality rates ranged respectively from 83% to 88% demonstrating that infections with MARV could be as severe as those caused by highly pathogenic species of EBOV. Sporadic cases of Marburg HF were reported in 2007 from Uganda [16]. In 2007 a non-fatal case was recognized in the United States [17], and a fatal case occurred in 2008 in the Netherlands [18]; both cases were imported from Uganda.

For a long time the epidemiological circumstances surrounding filovirus outbreaks suggested that bats may have served as the primary source of infection in humans and non-human primates. Before shipment from Uganda, the African green monkeys associated with the 1^st^ outbreak of Marburg HF in Europe in 1967, had been collected on the shores of Lake Victoria where they may have encouterd fruit bats [11]. In the 2^nd^ Marburg outbreak in 1975, Marburg HF first developed in one of the Australian tourists who had slept in rooms with insectivorous bats at two locations in Zimbabwe and had visited the Chinhoyi caves where bats may also have been present; subsequently the second tourist, and a nursing sister who had cared for the both patients, developed the disease [19]. The 1980 [11] and 1987 [12] fatal incidents of MARV HF were linked to entry into Kitum Cave on the Kenyan side of Mount Elgon, but the precise source of infection was not identified. It is intriguing that although Kitum Cave and similar caves are often easily accessible and frequently visited by tourists and local people, no futher cases of Marburg HF have been reported from Kenya. However, MARV RNA was detected in pooled liver, spleen and lung tissus of an apparently healthy, pregnant female *R. aegyptiacus* collected at Kitum Cave in July 2007 [20]. It has been speculated that the source of MARV in Kitum Cave might be transient rather than a permanent cave dweller or that virus replication in cave dwellers is restricted to tissues which do not allow for profuse viral shedding and extensive lateral spread. Alternatively, the absence of extensive MARV transmission to humans may be because only small numbers of the putative reservoir are infected at any one time. There is also the possibility, however slight, of rare mutations in filoviruses allowing for successful transmission from a reservoir to humans or non-human primates [21]. Despite intensive efforts to trap thousands of vertebrate and invertebrate hosts in filovirus outbreak areas, isolation of live EBOV or MARV from potential reservoirs was unsuccessful for a long time [22]–[25].

The first experimental inoculation study of insectivorous and fruit bats with EBOV conducted by Swanepoel et al. [22] demonstrated that infection with EBOV Zaire results in high viremia and virus replication in bat tissues but does not necessarily trigger clinical disease. The re-emergence of Ebola HF in the Congo basin in 1994, followed by repeated outbreaks in Gabon, Republic of Congo or DRC up to 2003, was associated with extensive mortalities in gorilla, chimpanzee and human communities [3], [5], [26]–[29]. In areas where non-human primates are rare or absent, hunting and eating of bats seem to have resulted in animal-to-human transmission of EBOV [4], [30], [31].

In the last two decades the association between MARV and bats was reinforced following transmission of the virus to humans that had encountered *R. aegyptiacus* in caves and mines. The 1998–2000 outbreak of Marburg HF in Durba, DRC was characterized by multiple occurrences of transmission in workers in Goroumbwa Mine, where large numbers of bats roosted [13], [32]. Repeated introductions of infection into humans from a natural source were supported by the identification of several genetic lineages of MARV shared by bats and humans during the outbreak. Diverse genetic lineages of MARV were detected in *R. aegyptiacus* and two species of insectivorous bats in the mine, *Rhinolophus eloquens* (the eloquent horse-shoe bat) and *Miniopterus inflatus* (the greater long-fingered bat). These bats and notably R. *aegyptiacus* also tested positive for specific antibodies to MARV [32]. Cessation of the outbreak coincided with flooding of the mine by 2000 [13]. The diversity of MARV sequences detected during the Durba epidemic suggests compartmentalized circulation of virus in large bat colonies or, alternatively, their involvement as intermediate hosts species. However, rodents, shrews, and various taxa of arthropods, including streblid, nycteriibid, and argasid ectoparasites of bats trapped at the mine were negative when tested for the virus [32]. EBOV RNA and specific antibodies were detected in fruit bats in Gabon during an ecological investigation which followed outbreaks of Ebola HF in humans and great apes [30]. The presence of MARV nucleic acid and specific antibodies were reported in *R. aegyptiacus* from Gabon in the absence of accompanying outbreaks of disease [33]. A recent serological survey showed cocirculation of EBOV and MARV in Gabonese bat populations, and a high prevalence of ELISA IgG antibodies to both viruses in *R. aegyptiacus* [34]. However, in 2007, the investigation of a small outbreak of Marburg HF in miners mining lead and gold in Kitaka Cave in western Uganda where large numbers of insectivorous and fruit bats were present, eventually led to the first isolation of live MARV from wild-caught and apparently healthy *R. aegyptiacus* [16]. The risk of transmitting filoviruses from bats to humans who enter bat habitats was reinforced again in December 2007 [17] and in July 2008 [18], when an American and a Dutch tourist respectively acquired MARV infection after encountering *R. aegyptiacus* in the Python Cave near Queen Elizabeth National Park, less than 25 miles from Kitaka mine. We present the virological and serological results of the first experimental infection of captive-bred *R. aegyptiacus* with MARV to the best of our knowledge, contributing significantly towards the establishment of an experimental bat-filovirus model.

## Materials and Methods

### *R. aegyptiacus* colony

**Figure 1:**
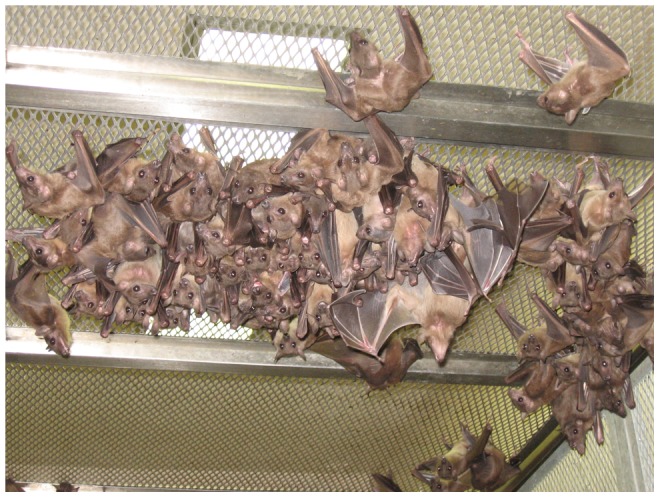
*R. aegyptiacus* colony maintained in large flight cage allowing for housing 150–200 bats.

Free-flying *R. aegyptiacus*, were captured by mist-net in the north-east of South Africa under a permit issued by the former Department of Environment Affairs (now Department of Agriculture, Conservation and the Environment) of the Limpopo Province local government, permit number 005–00002. Animal ethics approval was granted by the Animal Ethics Committee of the National Health Laboratory Service (NHLS), document number AEC 083/03, for colonization of the bats for research purposes. The bats were transported to the animal facility at NHLS in temporary cages and transferred to a large flight cage under BSL-2 containment for housing and colonization (Fig. 1). Blood samples were taken to confirm that the bats had not been exposed to rabies-related lyssaviruses or filoviruses prior to colonization. Colony conditions consisted of ambient temperatures ranging from 25–32°C and relative humidity >40% R.H. Bats were fed the equivalent of their own body mass of tropical fruits with banana forming the bulk of daily allocation and provided *ad libitum* fresh water in large, flat dishes (rim height of 2 cm; diameter of 40 cm) placed at floor level. Vitamin B-12 supplements were administered daily in the form of multi-vitamin syrup or powder added to the food and vitamin B-12 (Lennon™, Aspen-Pharmacare Ltd.) was injected intramuscularly at a dose of 1 µg per gram body mass twice a year. The cave-dwelling *R. aegyptiacus* adapted easily to the flight cage, which is constructed of stainless steel mesh on a stainless steel frame (Fig. 1). The room which houses the flight cage is temperature controlled and air-conditioned. The air-handling system consists of a fresh, partially-filtered air supply and a HEPA-filtered exhaust, with heating supplied by steam calorifier. Ambient humidity in Johannesburg is approximately 35% R.H. so that the added humidity is derived from the water spray used to clean the cage and room. The animal lab is cleaned daily with pressurized tap water at a temperature of approximately 50°C. Waste water from the cleaning process is passively treated in 2 serially-linked underground settling tanks before discharge into the municipal sewerage system.

### Accommodation and handling of *R. aegyptiacus* in BSL4

**Figure 2:**
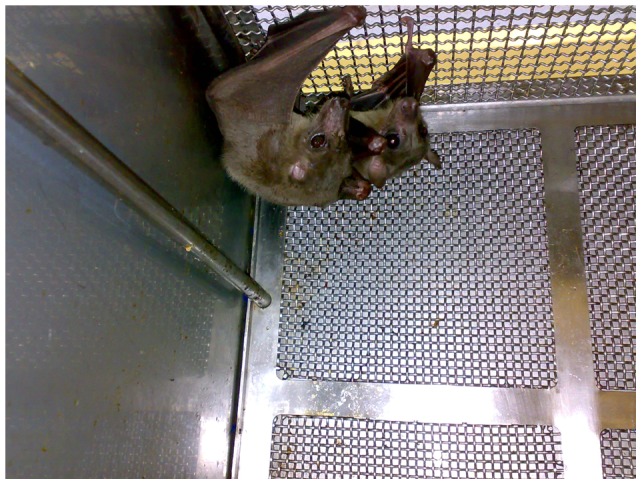
Custom-made experimental bat cage, which allows for housing 2–4 bats.

The animal husbandry and study design were endorsed by the Animal Ethics Committee of the NHLS, clearance number AEC 130/11. Captive-bred bats were housed in custom designed stainless steel cages with squeeze-backs and removable trays and mesh floors in groups of 2–4 per cage (Fig. 2). Cages were isolated under negative pressure in ventilated cabinets (Techniplast) with HEPA-filtered ventilation inside a single animal room within the biosafety level 4 (BSL4) facility housed at the National Institute for Communicable Diseases (NICD) of the NHLS. Room temperature was maintained at 22±1°C and a negative pressure of -66 kPa with 18 air changes per hour and humidity between 50–70% R.H. The bats were acclimatized to the BSL-4 environment for one week before the experimental procedures started. A total of 30 bats were used, consisting of 7 pups still attached to their mothers, 16 females and 7 males. Bats were fed fresh banana and provided *ad libitum* fresh water daily as for the colony.

### Viral inoculum

The Hogan isolate of MARV used to inoculate bats was originally isolated from the kidney of an Australian tourist who contracted a fatal disease in 1975 in Zimbabwe [10]. Animals were inoculated with Vero cells (passage # 38) cell culture supernatant containing 10^4^ TCID_50_ of MAR-MHK per ml.

### Experimental design

Bats were initially divided into 2 experimental groups, A and B. Animals were anaesthetized prior to inoculation and specimen collection using a mixture of ketamine HCl (Anaket-V®, Bayer (Pty) Ltd, South Africa) in a dose of 35 mg/kg body mass and xylazine (Rompum®, Bayer (Pty) Ltd. South Africa) in a dose of 5 mg/kg body mass given intramuscularly. Nine bats of group A (5 adult females, 1 pup, 3 adult males) were exposed to MAR-MHK oronasally by dripping 50 µl of inoculum into each nostril and 100 µl on the dorsal surface of the tongue. Group B consisting of 20 bats (adult females, pup, and adult males) was given 100 µl inoculum per bat by both intraperitoneal (i.p.) and subcutaneous (s.c.) route. Bats were monitored daily for the development of clinical signs and food intake. Feces and urine were collected daily from non-absorbent lining (Benchkote^TM^, Whatman®, G.E. Healthcare) in the tray of the cage until the end of the experiment. In addition rectal and oral swabs were taken on days 9 and 21 post inoculation (p.i.). Oral and rectal swabs were collected by using sterile cotton swabs and transferred to 0.5 ml of EMEM immediately. Blood and tissue samples were collected on days 2, 5, 9, and 21 p.i., with additional blood samples taken on days 7 and 16 p.i. On the day of the post mortem, bats were anaesthetized and then killed by cardiac exsanguination. At postmortem a wide range of tissues were collected for virological analysis. Occasionally urine samples were collected directly from the bladder by aspiration with a syringe.

The schedule for sample collection is given in Table 1. Animals were intensively sampled at the beginning and at the end of the experiment in order account and obtain evidence for acute infection and possible persistent infection, especially in the reproductive tract. Because no evidence of viral replication or seroconversion could be found in bats from group A, three remaining animals from this group were inoculated subcutaneously with 100 µl of the inoculum and became experimental group C (Table 1). Blood from this group was collected on day 5, and on day 8 p.i. all 3 bats were euthanized and blood and tissues collected for laboratory testing.

**Table 1:** Experimental design and sample collection schedule in *R. aegyptiacus* bats inoculated with Hogan strain of MARV.

Day p.i.	Day 2	Day 5	Day 7	Day 9	Day 16	Day 21
Group A	Blood and tissues†	Blood and tissues†	Blood^#^	Blood and tissues†	Blood^#^	Blood^#^
(n = 9)	1× male	1× male	1× male	1× female	1× male	1× male
Oral and nasal inoculation	1× female	1× female	3× females	1× pup	2× females	2× females
				Oral and rectal swabs		
				1x female		
Group B	Blood and	Blood and	Blood^#^	Blood and	Blood^#^	Blood and
(n = 21)	tissues†	tissues†		tissues†		tissues†
i.p.^a^ and s.c.^b^	1× male	1× male	3× males	2× females	3× males	3× males
inoculation	2× females	2× females	6× females	1× pup	4× females	4× females
	1× pup	1× pup				3× pups
				Blood^#^		Oral and
				3× males		rectal swabs
				2× females		1× male
						3× females
				Oral and rectal swabs		
				3× males		
				4× females		

Day p.i.	Day 5	Day 8
Group C	Blood^#^	Blood and
(n = 3)*		tissues†
s.c.	1× male	1× male
inoculation	2× females	2× females

a =  intraperitoneal.

b =  subcutaneous.

† =  lethal sampling.

# =  non-lethal sampling.

* =  three remaining bats from group A were later used as group C for s.c. inoculation.

Blood collected into EDTA tubes was centrifuged at 2000 rpm for 10 min and the resulting plasma aliquoted in equal volumes for storage at −70°C until testing, except for one aliquot which was processed for and tested by q-RT-PCR on the day of collection. Blood collected into untreated tubes was left in the refrigerator to clot and was either processed for serum on the day of collection or on the next day. Serum aliquots were stored at −70°C until used. Each tissue was aseptically subdivided, and its portions placed neat in cryotubes for virus isolation, and into RNA later (Qiagen) for q-RT-PCR. Neat tissue samples for virus isolation were stored at −70°C until used. Liver, spleen and lung tissues were processed for and tested by q-RT-PCR on the day of collection, the remaining tissue samples preserved in RNA later were stored at −70°C until used.

### Real-time quantitative RT-PCR (q-RT-PCR)

Approximately 100 mg of tissue collected into RNAlater was transferred into Eagles Minimal Essential Medium (EMEM) containing L-glutamine, non-essential amino acids, and antibiotics. Tissues were homogenized as 10% (w/v) suspensions at 30Hz for 8 minutes using a Tissuelyser II and stainless steel beads (Qiagen). Feces and urine pools were homogenized using the urine as diluent and processed as described for tissues. Oral and rectal swabs were mixed vigorously by vortexing for 30 seconds before centrifugation. All sample suspensions were centrifuged at 13,000 rpm for 5 minutes, and viral RNA was extracted from the resulting supernatants using QIAamp Viral RNA Mini Kit (Qiagen) according to the manufacturer's instructions. A 140 µl aliquot of clarified tissue and swabs suspensions, and serum was transferred to 560 µl AVL-lysis buffer, followed by automated viral RNA extraction using a QIAcube (Qiagen). RNA was eluted with 60 µl AVE elution buffer and stored at −70°C until testing. A 5 µl RNA aliquot was used to perform real-time quantitative RT-PCR (q-RT-PCR) with the Qiagen OneStep RT-PCR kit (Qiagen), using a 25ul total reaction volume. Primers FiloA2.3 and Filo B-Ra detecting the L gene were used according to the protocol published by Panning et al. [35] except for using a 0.1 µM concentration of the FAMMBG probe. The MARV broadly reactive RT-PCR assay detecting the VP40 gene as described by Towner et al. [14] using 0.5 µM concentrations of the primers and a 0.2 µM concentration of the probe was additionally performed on selected samples. Samples with C_t_ values ≤40 were regarded as positive. A quantification standard was generated by cloning the PCR target region into a pCRII-TOPO expression vector (Invitrogen). Inserts together with the Sp6 promoter sequence were amplified using vector specific universal M13 primers. In vitro transcription and DNase digestion was performed using the Megascript Sp6 kit (Ambion) according to the manufacturer's instructions. In vitro transcribed RNA was purified using an RNeasy mini kit (Qiagen), quantified spectrophotometrically and used as quantification standard and as a positive control. Other internal controls included an extraction control and no template control. Copy numbers of MARV RNA detected per reaction volume were converted to copy numbers per milliliter of plasma or gram of tissue. For extrapolation of RNA copy numbers into TCID_50_, stock MARV-MHK was log_10_ diluted and RNA extracts of each dilution subjected to q-RT-PCR testing in duplicate. A logarithmic titration curve generated (TCID_50_/ml:RNA copies/ml) was used for converting RNA copy numbers detected in samples tested into TCID_50_.

### Virus isolation and titration

Virus isolation was attempted on all blood and tissues as well as on all feces/urine pools, oral and rectal swabs. Supernatants of homogenated feces/urine pools were filtered through 0.22 µm syringe filters before inoculation. Approximately 100 mg of neat tissue was homogenized as 10% (w/v) suspensions using the same method as for processing tissues for q-RT-PCR testing. The isolation procedure followed the method described by Towner el al. [16]. Briefly, Vero E6 cells at 80–90% confluency in 25 cm^2^ tissue culture flasks (Corning) were inoculated with 500 µl of each sample and incubated for 1 hour at 37°C with occasional rocking. After incubation homogenates were discarded, Vero cells monolayers washed with sterile phosphate buffered saline (PBS) and fresh EMEM with antibiotics and 2% fetal calf serum was added. Inoculated flasks were incubated at 37°C for 14 days and medium was changed at 7 days p.i. Cultures were tested for MARV virus replication by q-RT-PCR using tissue culture fluids collected at the time of first visible cytopathic effect (CPE) or at 14 days p.i.

To correlate viral loads in plasma to extrapolated TCID_50_ values in selected samples, standard virus titrations were carried out in tissue culture grade 96-well microtitre plates (results not shown). A 100 µl aliquot of tenfold dilutions of plasma (starting from 1∶10) in EMEM was inoculated into each of four microtitre plate wells and 100 µl of Vero cell suspension containing 2×10^5^ cells/ml added to each well. The final concentration of foetal-calf serum in the total volume (200 µl) of the mixture was 4%. The inoculated microplates were incubated at 37°C in 5% CO_2_ and observed microscopically for CPE for 14 days after inoculation. Where CPE was unclear or unapparent, supernatants from the wells were subjected to RNA extraction and q-RT-PCR as described above to confirm replication of virus. Virus concentrations, calculated by the method of Kärber [36] were expressed as TCID_50_/ml of plasma.

### Serology

An enzyme-linked immunosorbent assay (ELISA) for the detection of immunoglobulin G (IgG) against MARV in bat sera or plasma was done as described previously (Swanepoel et al., 2007), except for using *R. aegyptiacus* positive and negative sera as internal controls. Net ELISA optical density values were expressed as percent positivity of a *R. aegyptiacus* positive control serum. Detection of neutralizing antibodies was attempted by virus neutralization test (VNT) with or without an addition of guinea pig complement using the procedure described by Fukunaga et al. [37] with some modifications. Bat sera were inactivated for 30 minutes at 56°C. Two sets of duplicate two-fold serial dilutions of each serum (from 1∶4 to 1∶512) were prepared in 25 µl EMEM on 96-well microtitre plates. Twenty five µl of EMEM was added to the first set of duplicate serum dilutions, and 25 µl of 10% guinea pig serum was added to the second set. Then 50 µl of EMEM containing 100 TCID_50_ of MARV-MHK was added to each well, and the virus-serum and virus-serum-complement mixtures were incubated for 60 minutes at 37°C in 5% CO_2_. After incubation, 100 µl of Vero cell suspension containing 2×10^5^ cells/ml of EMEM with 8% foetal calf serum was added to each well. Plates were incubated at 37°C in 5% CO_2_ and microscopically observed for CPE at regular intervals for the duration of 10 days. Neutralizing antibody titres were recorded as the reciprocal of the highest serum dilution inhibiting ≥75% of the CPE in both replicates.

### Statistical analysis

Statistical significances of differences in viral load levels and humoral immune responses were calculated by using the Fisher F test that calculates the two-tailed probability that variances in two arrays are not significantly different (Excel, Microsoft Office 2007). *P*-values ≤0.05 indicates a statistically significant difference.

## Results

### Clinical and post mortem observation

All bats remained clinically well and maintained normal food uptake throughout the period of the study, irrespective of inoculation route. None of the mothers abandoned their pups, an indication of the lack of stress, because of viral infection or other factors in these bats. No gross abnormalities or lesions were identified on postmortem examination.

### Detection of MARV-MHK by q-RT PCR and virus isolation

**Figure 3:**
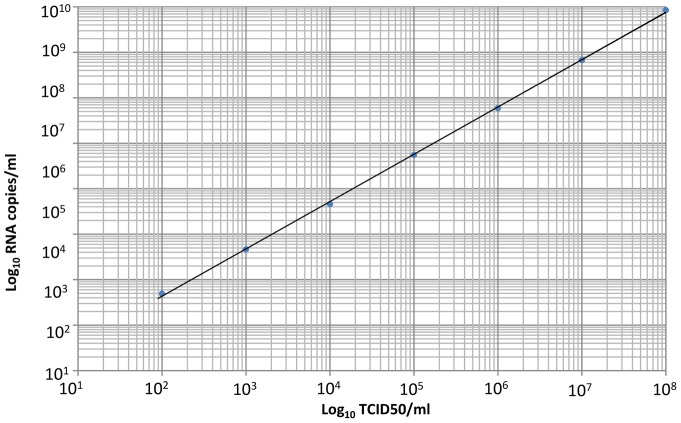
Correlation between MARV RNA copies versus TCID_50_ on a logarithmic scale graph (R^2^ = 0.999).

q-RT PCR performed on RNA extracts from log_10_ dilutions of stock MARV yielded a linear correlation between RNA copy numbers and TCID_50_ (*R^2^* = 0.999) (Fig. 3); 1 TCID_50_/ml of virus is equal to 5.86 RNA copies/ml.

No viremia or presence of MARV RNA could be detected in various tissues collected from 9 bats inoculated by a combination of nasal or oral routes (group A -results not shown).

**Figure 4:**
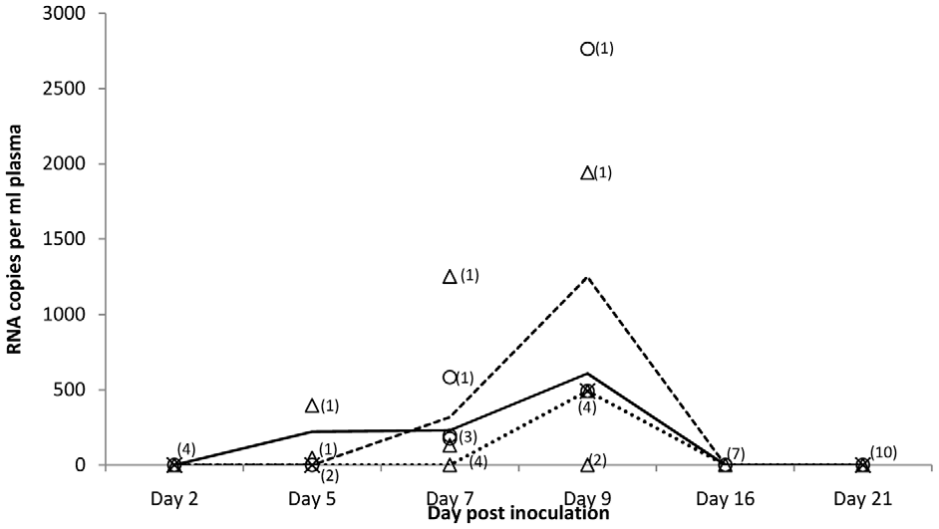
Viremia levels in *R. aegyptiacus* inoculated by intraperitoneal and subcutaneous route with MARV isolate MHK. MARV RNA copies per ml of plasma in individual adult females (Δ), adult males (○) and pups (x), and mean copy numbers for females (-----), males (---) and pups (•••) are shown.

In bats inoculated by a combination of subcutaneous and intraperitoneal route (group B), viremia lasted for at least five days; MARV was detected in plasma on days 5, 7 and 9 p.i., but not on day 16 p.i. (Fig. 4). The average level of viremia ranged in males from 10^3.3^ TCID_50_/ml on day 7 to 10^3.9^ TCID_50_/ml on day 9 p.i. and in females it was 10^3.1^ TCID_50_/ml on day 5, 10^3.6^ TCID_50_/ml on day 7 and 10^3.9^ TCID_50_/ml on day 9 p.i. In one pup, 10^3.5^ TCID_50_/ml was detected in plasma on day 9 p.i. In this experimental group MARV was regularly detected in liver (ranging from 10^4.8^ to 10^6.5^ TCID_50_/g tissue), spleen (10^3.2^ to 10^6.0^ TCID_50_/g tissue), intestine (10^3.7^ to 10^5.1^ TCID_50_/g tissue), bladder (10^4.4^ to 10^4.7^ TCID_50_/g tissue), and female reproductive tissues (10^5.7^ to 10^7.1^ TCID_50_/g tissue). Virus was occasionally detected in lung, heart, kidney, and salivary gland tissues with viral loads ranging from 10^3.7^ to 10^5.6^ TCID_50_/g tissue. MARV was also detected in the mammary gland tissues collected of a single female on day 9 p.i. (Table 2) where it replicated to 10^4.7^ TCID_50_/g tissue. Five discrepant results were noted between the PCR and virus isolation in group B; four specimens positive on virus isolation were negative by PCR, and one specimen negative on virus isolation was positive by PCR. Of the total of 283 blood and tissue samples tested in this group 33 (11.7%) were PCR positive and 36 (12.7%) were virus isolation positive (Table 2).

**Table 2:** PCR and virus isolation results in *R. aegyptiacus* bats inoculated subcutaneously and intraperitoneally with Hogan strain of MARV.

	Females (n = 10)				Males (n = 5)				Pups (n = 6)			
Sample type	Days post inoculation				Days post inoculation				Days post inoculation			
	2	5	9	21	2	5	9	21	2	5	9	21
Plasma	PCR 0/2 VI 0/2	**PCR 2/2 VI 2/2**	**PCR 2/2 VI 2/2**	PCR 0/4 VI 0/4	PCR 0/1 VI 0/1	PCR 0/1 VI 0/1	**PCR 3/3 VI 3/3**	PCR 0/3 VI 0/3	PCR 0/1 VI 0/1	PCR 0/1 VI 0/1	**PCR1/1 VI 1/1**	PCR 0/3 VI 0/3
Liver	PCR 0/2 VI 0/2	**PCR 2/2 VI 2/2**	**PCR 1/2 VI 1/2**	PCR 0/4 VI 0/4	PCR 0/1 VI 0/1	PCR 0/1 VI 0/1	n.s.	PCR 0/3 VI 0/3	PCR 0/1 VI 0/1	**PCR1/1 VI 1/1**	PCR 0/1 VI 0/1	PCR 0/3 VI 0/3
Spleen	**PCR 1/2 VI 1/2**	**PCR 2/2 VI 2/2**	**PCR 2/2 VI 2/2**	PCR 0/4 VI 0/4	PCR 0/1 VI 0/1	PCR 0/1 VI 0/1	n.s.	PCR 0/3 VI 0/3	PCR 0/1 VI 0/1	**PCR1/1 VI 1/1**	PCR 0/1 VI 0/1	PCR 0/3 VI 0/3
Kidney	PCR 0/2 VI 0/2	PCR 0/2 VI 0/2	PCR 0/2 VI 0/2	PCR 0/4 VI 0/4	PCR 0/1 VI 0/1	PCR 0/1 VI 0/1	n.s.	PCR 0/3 VI 0/3	PCR 0/1 VI 0/1	**PCR1/1 VI 1/1**	PCR 0/1 VI 0/1	PCR 0/3 VI 0/3
Lung	PCR 0/2 VI 0/2	PCR 0/2 VI 0/2	**PCR 0/2 VI 1/2**	PCR 0/4 VI 0/4	PCR 0/1 VI 0/1	PCR 0/1 VI 0/1	n.s.	PCR 0/3 VI 0/3	PCR 0/1 VI 0/1	**PCR 1/1** VI 0/1	PCR 0/1 VI 0/1	PCR 0/3 VI 0/3
Heart	PCR 0/2 VI 0/2	**PCR 1/2 VI 1/2**	PCR 0/2 VI 0/2	PCR 0/4 VI 0/4	PCR 0/1 VI 0/1	PCR 0/1 VI 0/1	n.s.	PCR 0/3 VI 0/3	PCR 0/1 VI 0/1	PCR 0/1 VI 0/1	**PCR1/1 VI 1/1**	PCR 0/3 VI 0/3
Reproductive tract	PCR 0/2 VI 0/2	**PCR 1/2 VI 1/2**	**PCR 2/2 VI 2/2**	PCR 0/4 VI 0/4	PCR 0/1 VI 0/1	PCR 0/1 VI 0/1	n.s.	PCR 0/3 VI 0/3	n.s.	n.s.	n.s.	n.s.
Salivary glands	PCR 0/2 VI 0/2	**PCR 1/2 VI 1/2**	PCR 0/2 VI 0/2	PCR 0/4 VI 0/4	PCR 0/1 VI 0/1	PCR 0/1 VI 0/1	n.s.	PCR 0/3 VI 0/3	PCR 0/1 VI 0/1	PCR 0/1 VI 0/1	PCR 0/1 **VI 1/1**	PCR 0/3 VI 0/3
Intestine	PCR 0/2 VI 0/2	**PCR 1/2 VI 1/2**	**PCR 1/2 VI 2/2**	PCR 0/4 VI 0/4	PCR 0/1 VI 0/1	PCR 0/1 VI 0/1	n.s.	PCR 0/3 VI 0/3	PCR 0/1 VI 0/1	PCR1/1 VI 1/1	**PCR1/1 VI 1/1**	PCR 0/3 VI 0/3
Bladder	PCR 0/2 VI 0/2	**PCR 1/2 VI 1/2**	**PCR 1/2 VI 2/2**	PCR 0/4 VI 0/4	PCR 0/1 VI 0/1	PCR 0/1 VI 0/1	n.s.	PCR 0/3 VI 0/3	PCR 0/1 VI 0/1	PCR 0/1 VI 0/1	**PCR1/1 VI 1/1**	PCR 0/3 VI 0/3
Mammary gland	n.s.	n.s.	**PCR 1/1 VI 1/1**	PCR 0/4 VI 0/4	n.s.	n.s.	n.s.	n.s.	n.s.	n.s.	n.s.	n.s.
Muscle	PCR 0/2 VI 0/2	PCR 0/2 VI 0/2	PCR 0/2 VI 0/2	PCR 0/4 VI 0/4	PCR 0/1 VI 0/1	PCR 0/1 VI 0/1	n.s	PCR 0/3 VI 0/3	PCR 0/1 VI 0/1	PCR 0/1 VI 0/1	PCR 0/1 VI 0/1	PCR 0/3 VI 0/3
Skin	PCR 0/2 VI 0/2	PCR 0/2 VI 0/2	PCR 0/2 VI 0/2	PCR 0/4 VI 0/4	PCR 0/1 VI 0/1	PCR 0/1 VI 0/1	n.s	PCR 0/3 VI 0/3	PCR 0/1 VI 0/1	PCR 0/1 VI 0/1	PCR 0/1 VI 0/1	PCR 0/3 VI 0/3
Brain	PCR 0/2 VI 0/2	PCR 0/2 VI 0/2	PCR 0/2 VI 0/2	PCR 0/4 VI 0/4	PCR 0/1 VI 0/1	PCR 0/1 VI 0/1	n.s	PCR 0/3 VI 0/3	PCR 0/1 VI 0/1	PCR 0/1 VI 0/1	PCR 0/1 VI 0/1	PCR 0/3 VI 0/3

PCR  =  PCR results.

VI  =  Virus isolation results.

(/)  =  Values in brackets indicate number of positive bats per number of animal tested at a specific time point by PCR and VI.

n.s.  =  Not sampled.

In group C, virus was detected in plasma (10^2.4^ to 10^3.3^ TCID_50_/ml), liver (10^3.9^ to 10^4.3^ TCID_50_/g tissue) and spleen (10^3.6^ to 10^4.0^ TCID_50_/g tissue) in two out of three inoculated bats. No virological evidence (PCR and virus isolation negative) of infection could be found in the third bat (female), but serological monitoring for this group was of very short duration. On one occasion MARV was detected in the female reproductive tissues (Table 3) where it replicated to 10^3.9^ TCID_50_/g tissue. Two discrepant results were noted between the PCR and virus isolation in group C; two specimens positive on virus isolation were negative by PCR. Of the total of 42 blood and tissue samples tested in this group 8 (19%) were PCR positive and 10 (23.87%) were virus isolation positive (Table 3). The higher detection rate of MARV by virus isolation suggests that the q-RT PCR might be less sensitive for the detection of Vero-cell adapted Hogan strain in bat tissues. The discrepancies between the q-RT PCR and virus isolation noted in this study could be also due to some intrinsic properties of the two detection methods used, including assay tolerance for inhibitory factors and different volumes of tissues subjected for testing. In the context of the latter, the chance to detect lower concentration of virus might be increased by testing larger volumes of infected tissues by Vero cells inoculation.

**Table 3:** PCR and virus isolation results in *R. aegyptiacus* bats inoculated subcutaneously with Hogan strain of MARV.

	Females (n = 2)		Males (n = 1)	
Sample type	Days post inoculation		Days post inoculation	
	5	8	5	8
Plasma	**PCR 1/2 VI 1/2**	**PCR 1/2 VI 1/2**	**PCR 1/1 VI 1/1**	PCR 0/1 **VI 1/1**
Liver	n.s.	**PCR 1/2 VI 1/2**	n.s.	**PCR 1/1 VI 1/1**
Spleen	n.s.	**PCR 1/2 VI 1/2**	n.s.	**PCR 1/1 VI 1/1**
Kidney	n.s.	PCR 0/2 VI 0/2	n.s.	PCR 0/1 VI 0/1
Lung	n.s.	PCR 0/2 VI 0/2	n.s.	PCR 0/1 VI 0/1
Heart	n.s.	PCR 0/2 VI 0/2	n.s.	PCR 0/1 VI 0/1
Reproductive tract	n.s.	**PCR 1/2 VI 1/2**	n.s.	PCR 0/1 VI 0/1
Salivary glands	n.s.	PCR 0/2 VI 0/2	n.s.	PCR 0/1 VI 0/1
Intestine	n.s.	PCR 0/2 VI 0/2	n.s.	PCR 0/1 VI 0/1
Bladder	n.s.	PCR 0/2 **VI 1/2**	n.s.	PCR 0/1 VI 0/1
Muscle	n.s.	PCR 0/2 VI 0/2	n.s.	PCR 0/1 VI 0/1
Skin	n.s.	PCR 0/2 VI 0/2	n.s.	PCR 0/1 VI 0/1
Brain	n.s.	PCR 0/2 VI 0/2	n.s.	PCR 0/1 VI 0/1

PCR  =  PCR results.

VI  =  virus isolation results.

(/)  =  values in brackets indicate number of positive bats per number of animal tested at a specific time point by PCR and VI.

n.s.  =  not sampled.

Virus was not detected in muscle, skin or brain tissues collected after cardiac exsanguination in both groups B and C (Table 2 and 3), indicating that blood did not contribute to positive results in other tissues. The presence of MARV was not detected in extracts from feces and urine and rectal swabs additionally subjected to the RT-PCR detecting the VP 40 gene.

### Serology

**Figure 5:**
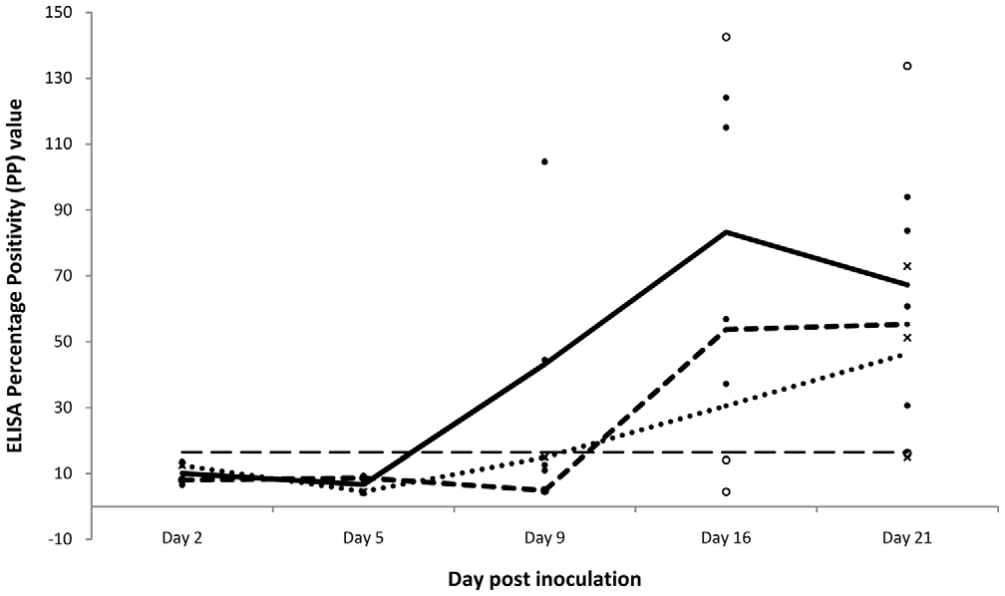
IgG responses in *R. aegyptiacus* bats inoculated with MARV isolate MHK. Individual responses for adult females (•), adult males (○) and pups (x), and mean responses for females (----------), males (----------) and pups (•••) are shown. The ELISA cut-off value is indicated as a dashed line (---).

IgG anti-MARV antibody was first detected on day 9 p.i.in two females. By day 21 p.i. most test animals had seroconverted with the highest mean IgG antibody response recorded in females and the lowest in pups (Fig. 5). However, except for day 9 p.i. (p = 0.005), statistically there was not a significant difference in IgG responses between females, males and pups. There was only a slight increase in mean IgG antibody levels in males and pups between days 16 and 21 p.i., compared to its decrease in females. Virus neutralization antibody titers ranging from 1∶4 to 1∶8, were detected in 3 out of 9 bat sera collected on day 21 post inoculation in both VNT with and without addition of complement. The inclusion of 10% guinea pig serum in virus-serum mixture did not significantly increased VNT antibody titers. However, two sera having titers of 1∶4 in VNT without 10% guinea pig complement had one dilution higher titers (1∶8) in VNT with an addition of complement.

## Discussion

It has been postulated that filoviruses are maintained in nature by transmission between bats by intra-specific aggressive, grooming and reproductive behaviour or through ectoparasites vectors [21]. Under this scenario, filovirus transmission from bats to humans would then occur either by direct contact with infected bats, including processing of their meat for consumption or might result from consumption of fruit contaminated with blood, urine, feces or placentas during parturition of infected bats [22], [30], [34]. It is generally accepted that if a bat is capable of circulating a virus for a prolonged period of time without clinical disease then the species may be suspected to be a reservoir host. In this study *R. aegyptiacus* inoculated with MARV became viremic, developed systemic infection, but remained clinically normal; no gross pathology was identified on post mortem examination. Similar results were reported in insectivorous bat species, *Mops condylurus*, the Angolan free-tailed bat and *Chaerephon pumilus*, the little free-tailed bat, and an epauletted fruit bat, *Epomophorus wahlbergi* experimentally infected with EBOV Zaire [22]. Likewise in humans, non-human primates, and other susceptible animals, MARV could be detected in various tissues of experimentally infected *R. aegyptiacus*, including liver and spleen tissues which are targeted sites in highly susceptible species. Known target cells that support filovirus replication in both natural host and experimental animal models include monocytes, macrophages, dendritic cells (DCs), hepatocytes, adrenal cortical cells, fibroblast and endothelial cells [38]–[48]. Monocytes, macrophages, and DCs not only regulate innate and adaptive immune responses but are also early targets of filovirus infection and possibly contribute to rapid and widespread dissemination of filoviruses in an infected host [42], [48]–[53]. The structure of sinusoids and sinuses in the liver and spleen allows for the direct migration of filoviruses from the blood stream, facilitating the infection of hepatocytes and splenic macrophages. Therefore, it appears that filoviruses are disseminated in the infected host by multiple mechanisms including transport of free virus particles by blood and lymphatic system, migration of infected monocytes and DCs into various tissues, and cell-to-cell spread via cell protrusions [6]. Results of our study indicate that the mechanism of viral dissemination and the pantropic nature of MARV infection in *R. aegyptiacus* might be similar to what is seen as in highly susceptible vertebrate hosts. However, the molecular and immunological mechanisms by which *R. aegyptiacus* counteracts the pathogenic effects of MARV replication observed in humans and non-human primates remains to be investigated. The consistently demonstrated presence of MARV in liver and spleen of experimentally inoculated *R. aegyptiacus* correlates with the detection of MARV nucleic acid in these tissues from naturally infected *R. aegyptiacus* [3], [16].

Neutralizing antibodies irrespective of the presence or absence of complement were rather low and not detected in all inoculated animals. Thus likewise in humans and non-human primates, neutralizing antibodies possibly do not render a protective mechanism against MARV replication in *R. aegyptiacus* [54]. However, in our experiments immune responses were not followed up for a long period and thus the ability of infected bats to develop neutralizing antibodies against MARV requires further study. It would be of great importance to know whether humoral responses in bats to MARV as measured by ELISA are long-lasting or only transient.

MARV was detected in *R. aegyptiacus* tissues which could be implicated in horizontal transmission, e.g. lung, intestines, kidney, bladder, and salivary glands. However, these tissues did not support virus replication to high titers for a prolonged time, and MARV was not detected in feces, urine, nasal or oral swabs. Consequently the mechanism of transmission between *R. aegyptiacus* bats and from this species to other animals remains unresolved. Nasal and oral swabs were taken only on two occasions thus one can argue that the virus could be missed if shedding was intermittent. In the study by Towner et al. [16] MARV RNA was also not detected in oral swabs taken from bats, including those with virus RNA-positive liver and spleen samples, suggesting that MARV transmission to other animals via masticated fruit pulp is unlikely. One cannot exclude the possibility that the virus loads in nasal secretions and saliva were below the detection limits of the assays used. *R. aegyptiacus* is a cave-roosting species, occurring in colonies of up to thousands, implying that even intermittent or very low shedding might sustain respiratory or oral spread of infection between bats in confined spaces. It has been postulated that increased viral shedding might be induced by stress or immunosuppression. However, unsuccessful infection of bats by the nasopharyngeal route suggests that these mechanisms of spread do not constitute the primary mode of MARV transmission in *R. aegyptiacus.* The apparent inefficiency of respiratory spread of MARV in this species appears to be further supported by a recent investigation of a Marburg HF outbreak associated with a large colony of *R. aegyptiacus* roosting in Kitaka Cave in Uganda [16].

It is also noteworthy that the sporadic presence of MARV in salivary glands and lung was only detected in bats inoculated by a combination of intraperitoneal-subcutaneous route, but not in bats inoculated subcutaneously. Intraperitoneal inoculation might find some future application for a bat-filovirus experimental model, but it does not mimic natural exposure to the virus. Similarly virus presence in intestine, kidney and bladder was only detected in bats inoculated by a combination of intraperitoneal-subcutaneous route, but not in bats inoculated subcutaneously. Successful recovery of EBOV from feces of *E. wahlbergi* on day 21 post inoculation led to the hypothesis that fruit bat excreta (contamination of food) might provide a source of filovirus transmission to humans and apes [4], [30]. Results from our study do not support this assumption; despite daily sampling of urine and feces up to 21 days after inoculation, *R. aegyptiacus* excreta remained negative for the presence of MARV.

In our study, MARV virus was often detected in blood, liver, spleen and the female reproductive tract but not in the male reproductive tract. Although not significantly prolonged as is a case for a number of arboviral infections, the duration of viremia demonstrated in *R. aegyptiacus*, would support transmission by hematophagous vectors or transmission of infection amongst *R. aegyptiacus* and to other vertebrate hosts by direct contact with infected blood. The colonies in which *R. aegyptiacus* bats are found in nature, combined with the vast areas covered by these bats in search of food and migration between different roosts from different geographical locations, could possibly sustain circulation of viruses, even if they only cause acute infection such as that shown in this study. This could mean that different roosts can be affected at different times, giving rise to persistence of virus in “patches” where infection disappears irregularly, only becoming infected again once a new generation of susceptible bats are available [55], [56]. In the experimental inoculation of EBOV in bats, Swanepoel et al. [22] demonstrated replication of the virus to high titers in the blood of both insectivorous and fruit bats. Turell at al. [57] failed to demonstrate replication of EBOV Reston in culicine and aedine mosquitoes and *Ornithodoros* ticks, but experimental inoculation of arthropods with more virulent species of EBOV were not undertaken. Kuntz et al. [58] reported that MARV could persist in *Aedes* mosquitoes for at least 3 weeks. Many potential blood-feeding arthropod vectors, including ticks and wingless flies specifically associated with bats have not been tested by experimental inoculation. During naturally acquired filovirus infections, both MARV and EBOV have been isolated from seminal fluid in human months after disease onset and full clinical recovery [11], [59], [60]. These findings led to the suspicion that prolonged filoviral infections or a delay in virus clearance from privileged sites may occur. We could not find any virological evidence of MARV presence in the *R. aegyptiacus* male reproductive tract three weeks after infection, but we could demonstrate MARV presence in the female *R. aegyptiacus* reproductive tract which implies that mother-to-pup transmission may occur. However, results of a recent study conducted by Towner et al. [16] in *R.aegyptiacus* juveniles and pregnant females wild-caught in Uganda seem to not support the possibility of mother-to-pup MARV transmission.

The MARV used in this study was recovered from a patient infected in Zimbabwe, and we therefore assume that this local virus isolate might have a better fitness to our colony of *R. aegyptiacus* originating from Limpopo province of South Africa. However, the susceptibility of different populations of *R. aegyptiacus* to different strains/isolates of the virus remains to be investigated. The passage history of MARV isolate used in this study may have a bearing on our results (e.g. on the severity of infection, level of viral replication and shading pattern), but the effect of *in vitro* passage history on genetic and biologic characteristics of filoviruses remains to be investigated.

The biology of a reservoir-filovirus system is notoriously poorly understood and much remains to be learned from *in vivo* reservoir model studies. This work confirms the susceptibility of *R. aegyptiacus* to infection with MARV irrespective of sex and age and contributes to establishing a *R. aegyptiacus*-filovirus experimental model. Further studies are required to uncover the mode of MARV transmission, and to investigate the putative role of *R. aegyptiacus* as a reservoir host.

### Ethics Statement

This study was carried out in strict accordance with the recommendations of the South African National Standards for the Care and Use of Animals for Scientific Purposes (SANS 10386∶2008). The protocols for establishment of a *Rousettus aegyptiacus* colony at NICD and the Marburg virus experimental infection study were approved by the National Health Laboratory Service Animal Ethics Committee (clearance numbers AEC 083/03 and AEC 130/11 respectively). Blood collection was done under Ketamine/Xylazine anaesthesia, and organ tissues collected from exsanguinated bats, and all efforts were made to minimize suffering.
